# Ovariotomy at the Samaritan Hospital

**Published:** 1878-05

**Authors:** 


Ovariotomy at the Samaritan Hospital.—In one of his last lectures in the Samaritan Hospital, before retiring from the active work of the institution, Mr. Spencer Wells gave a brief, but interesting review of the twenty years of his connection with the hospital. This includes the history of ovariotomy in England, from the first unsuccessful cases of Baker Brown, to the records of the Samaritan for the last two years, which show sixty-eight cases and sixty-one recoveries. During the first five years, about one case in three have died at the Samaritan; during the second and third five years, about one in four; in the fourth five years, about one in five, but in the last two only about one in ten died. The antiseptic system is now on trial, and Mr. Wells is more hopeful of favorable results since thymol has been introduced as a substitute for carbolic acid. Thymol has a pleasant odor, and has no poisonous properties, and, according to Dr. Burdon Sanderson, is a hundred times more effective as a germicide than carbolic acid. Volkmann has had encouraging results even with so weak a solution as one part in two thousand of water. The surgery of the hospital, however, has not been confined to ovariotomy, and a long list of operations is mentioned, the latest and most interesting of which are nephrotomy and the draining of a renal cyst, under strict antiseptic precautions. Mr. Wells proposes to hold public consultations at the hospital one day in the week, on a similar plan to the one followed at St. Bartholomew's Hospital, not wishing to sever himself entirely from the scenes of his labors and successes.—*British Medical Journal*, Dec. 15, 1877.

